# Personalizing Relapsing–Remitting Multiple Sclerosis Monitoring: Patient Acceptance of Serum Neurofilament Light Chain and the Role of Disease Knowledge

**DOI:** 10.3390/jpm16040185

**Published:** 2026-03-29

**Authors:** Ángel Pérez-Sempere, Elena García-Arcelay, Jacobo Caruncho Pérez, Antonio Candeliere-Merlicco, Aida Orviz, Jesús Martín-Martínez, Raquel Piñar-Morales, Elena Álvarez-Rodríguez, Eva M. Pacheco-Cortegana, Laura Borrega, Ignacio Casanova, Ana Belén Caminero, José Luis Sánchez-Menoyo, Montserrat Gómez-Gutiérrez, Olga Carmona, Carmen Calles, Miguel Ángel Hernández, Pablo López-Muñoz, Fabien Bakdache, Enric Monreal, Inés González-Suárez, Jorge Maurino

**Affiliations:** 1 Department of Neurology, Hospital Universitario General de Alicante, Universidad Miguel Hernández, 03010 Alicante, Spain; aperezs@mac.com; 2 Medical Department, Roche Farma, 28042 Madrid, Spain; elena.garcia_arcelay.eg1@roche.com; 3 AVEMPO (Asociación Viguesa de Esclerosis Múltiple de Pontevedra), 36208 Vigo, Spain; 4 Department of Neurology, Hospital Rafael Méndez, 30813 Lorca, Spain; candeliereantonio@gmail.com; 5 Department of Neurology, Hospital Universitario Fundación Jiménez Díaz, 28040 Madrid, Spain; aida.orviz@gmail.com; 6 Department of Neurology, Hospital Universitario Miguel Servet, 50009 Zaragoza, Spain; jmartinma@salud.aragon.es; 7 Department of Neurology, Hospital Universitario Clínico San Cecilio, 18007 Granada, Spain; rpinarmorales@gmail.com; 8 Department of Neurology, Hospital Universitario Álvaro Cunqueiro, 36312 Vigo, Spain; elena.alvarez.rodriguez@sergas.es (E.Á.-R.); igonsua@gmail.com (I.G.-S.); 9 Department of Neurology, Hospital Universitario Juan Ramón Jiménez, 21005 Huelva, Spain; evapachecocortegana@gmail.com; 10 Department of Neurology, Hospital Universitario Fundación Alcorcón, 28922 Madrid, Spain; laura.borrega@salud.madrid.org; 11 Department of Neurology, Hospital Universitario de Torrejón, 28850 Madrid, Spain; i.casanovap@gmail.com; 12 Department of Neurology, Complejo Asistencial de Ávila, 05071 Ávila, Spain; caminero.nrl@gmail.com; 13 Department of Neurology, Galdakao-Usansolo University Hospital, Osakidetza (Basque Health Service), 48960 Usansolo, Spain; sanchezmenoyo@yahoo.es; 14 Department of Neurology, Hospital San Pedro de Alcántara, 10003 Cáceres, Spain; montsegomezg@hotmail.com; 15 Department of Neurology, Fundació Salut Empordà, 17600 Figueres, Spain; occodina@gmail.com; 16 Department of Neurology, Hospital Universitari Son Espases, 07120 Palma, Spain; mcalles22@yahoo.es; 17 Department of Neurology, Hospital Nuestra Señora de la Candelaria, 38010 Tenerife, Spain; mhernandezp78@hotmail.com; 18 Department of Neurology, Hospital Llíria Arnau, 46160 Llíria, Spain; lopez_pabmun@gva.es; 19 Medical Department, Hoffmann-La Roche Limited, Mississauga, ON, ON L5N 5M8, Canada; fabien.bakdache@roche.com; 20 Department of Neurology, Hospital Universitario Ramón y Cajal, 28034 Madrid, Spain

**Keywords:** serum neurofilament light chain, multiple sclerosis, biomarker, patient preference, personalized medicine, shared decision-making

## Abstract

**Background**: Serum neurofilament light chain (sNfL) is an established biomarker of neuroaxonal damage in multiple sclerosis (MS). Despite its prognostic utility, patient awareness of its clinical application remains poorly characterized. The objective of this study was to assess the acceptance of sNfL monitoring among patients with early-stage relapsing–remitting MS (RRMS) and identify factors predicting their willingness to adopt this tool. **Methods**: This non-interventional, cross-sectional study was conducted across 16 neuroimmunology clinics. We included RRMS patients with a disease duration of ≤3 years receiving disease-modifying therapy. Acceptance was assessed following a standardized educational tutorial. Multivariable logistic regression was employed to identify predictors of patient acceptance. **Results**: The study included 144 patients (mean age 37.6 [SD 10.3] years, 69.4% female). Only 19.4% (*n* = 28) had prior awareness of sNfL. However, after the tutorial, 84.0% (*n* = 121) expressed willingness to adopt sNfL testing. Furthermore, 62.5% (*n* = 90) indicated that normal sNfL levels would provide emotional reassurance between clinical visits. Patients willing to undergo testing showed higher disease knowledge, less treatment regret, and better physical quality of life and cognitive performance. In the multivariable analysis, higher disease knowledge (OR = 1.52, 95%CI 1.16–1.99; *p* = 0.002) and lower symptom burden (OR = 0.96, 95%CI 0.93–0.99; *p* = 0.038) were associated with greater acceptance. **Conclusions**: Patients demonstrate high receptivity to sNfL monitoring when provided with adequate clinical context. Because disease knowledge is a primary driver of acceptance, personalized educational initiatives may be a complementary strategy to facilitate the integration of precision biomarkers into MS management.

## 1. Introduction

Despite the transformative introduction of high-efficacy disease-modifying therapies over the past decade, patients with multiple sclerosis (MS) continue to face significant uncertainty regarding their disease trajectory and long-term prognosis [1,2]. This uncertainty is a common phenomenon in MS, frequently manifesting as a fear of progression, and is correlated with psychological distress, including anxiety, depressive symptoms, and a lower quality of life [1,3,4]. Furthermore, high levels of uncertainty are reported by patients who have lower disease knowledge and poorer self-management abilities, highlighting a critical need for objective monitoring and enhanced education [1,5].

The field of neurology is increasingly integrating innovative fluid biomarkers to provide objective, quantifiable measures of disease activity [6,7,8]. Among these, serum neurofilament light chain (sNfL), a structural protein released into the cerebrospinal fluid and subsequently the blood following neuroaxonal damage, has emerged as a robust marker of neural injury across a broad spectrum of neurological disorders [7,8].

In the management of MS, sNfL functions as a clinically actionable biomarker that facilitates the detection of subclinical disease activity [8,9,10,11]. This neuroaxonal injury often precedes both clinical relapses and traditional changes detectable by means of magnetic resonance imaging (MRI) [5,12]. While clinical assessments and annual MRI scans remain the standard of care, these tools are temporally limited and may only identify damage after irreversible neurological injury has occurred. The sensitivity of sNfL allows clinicians to react more rapidly to poor disease control and facilitates timely therapeutic adjustments [8,9,10,11,13,14]. From the patient’s perspective, sNfL testing offers the potential to mitigate uncertainty by providing objective evidence of disease stability between routine follow-up visits. However, the successful integration of sNfL relies on robust scientific evidence but also equally on the perspectives and behaviors of patients and healthcare professionals [15,16,17]. While initial studies have begun to explore factors influencing neurologists’ openness to sNfL adoption, the patient perspective remains the least explored variable in the successful clinical integration pathway [18,19]. Therefore, the objective of this study was to assess the acceptance of sNfL testing among patients with early-stage relapsing–remitting MS (RRMS) and to identify the clinical, psychological, and behavioral factors associated with their willingness to adopt this new monitoring tool.

## 2. Methods

### 2.1. Study Design and Participants

This was an observational, cross-sectional study conducted at 16 hospital-based neuroimmunology clinics in Spain. Participants were enrolled consecutively during routine follow-up visits between 29 October 2024 and 4 April 2025. Every patient meeting the inclusion criteria, a diagnosis of RRMS, disease duration ≤ 3 years, and current use of disease-modifying therapy, was invited to participate in the study. This multicenter design was intended to capture clinical diversity across various regions in Spain. The study was approved by the research review board of Euskadi (CEIm-E), Spain. All participants provided written informed consent.

### 2.2. Study Procedure

Patient participation involved a single-visit, two-stage procedure using an electronic survey. Patients first completed an assessment of their prior sNfL awareness using a five-option Likert scale (Appendix A). Awareness was defined by the sum of participants who reported having some understanding or higher (Likert options 3, 4, and 5). Patients then received a brief educational tutorial that explained the mechanism of sNfL and its role in detecting ongoing disease activity (Appendix A). Immediately following the tutorial, the primary outcome, patient acceptance of sNfL testing, was assessed using the question *“After reading this information, would you accept the use of NfL in blood to monitor your disease activity?”* Responses to this 5-point Likert scale question were dichotomized for analysis as “willing to accept” (agreed/strongly agreed) versus “unwilling to accept” (neither agree nor disagree/disagree/strongly disagree) (Appendix A). The survey also assessed their perceived emotional reassurance (e.g., feeling more at ease).

### 2.3. Outcome Measures

All participants completed a comprehensive battery of patient-reported outcome measures and the Symbol Digit Modalities Test (SDMT) [20].

Disease knowledge: assessed using the Multiple Sclerosis Knowledge Assessment Scale (MSKAS) (22 items, true/false; higher scores indicate greater knowledge) [21]. This section also included the question: *“Has your long-term prognosis ever been discussed during your neurology appointments?”* to assess patient-physician communication regarding future disease trajectory.

Illness-related uncertainty: measured by the Mishel Uncertainty of Illness Scale (MUIS) (17-item scale, 5-point Likert scale; higher scores reflect greater uncertainty) [22].

Decisional Conflict: assessed using the Decisional Conflict Scale (SURE) (4 items; score < 4 indicates decisional conflict) [23].

Decision regret: measured by the Decision Regret Scale (DRS) (5 items, 5-point Likert scale; higher scores indicate greater regret related to a past treatment decision) [24].

Symptom severity: assessed using the SymptomMScreen questionnaire (SyMS) (12 items, 7-point Likert scale; higher scores reflect overall symptom severity) [25].

Quality of life: measured by the Multiple Sclerosis Impact Scale (MSIS-29) (29 items, Physical and Psychological Impact subscales; lower scores indicate higher health-related quality of life) [26].

Psychological impact: included the Hospital Anxiety and Depression Scale (HADS), the short-form State Anxiety subscale of the Spielberger State-Trait Anxiety Inventory (STAI) for anticipatory anxiety, and the Beck Hopelessness Scale (BHS) for measuring negative expectations regarding the future [27,28,29].

Cognitive Function: assessed using the SDMT. This test requires participants to match geometric symbols to corresponding numbers using a key [20]. The final score is the total number of correct substitutions completed within 90 s.

### 2.4. Statistical Analysis

Descriptive statistics were used for continuous (mean, standard deviation [SD], 95% confidence intervals [CI]) and categorical (frequencies and percentages) variables. Differences between the two groups (willing vs. unwilling to accept sNfL) were compared using independent samples *t*-tests or Mann-Whitney tests (continuous data) and Chi-square or Fisher’s exact tests (categorical data). Multivariable logistic regression analysis was performed to determine the association between patient characteristics and the primary outcome (willingness to accept sNfL testing). The statistical analysis was performed using Stata Statistical Software 17.0 (StataCorp., College Station, TX, USA).

## 3. Results

The study cohort consisted of 144 patients. The mean age was 37.6 (SD 10.3) years, with a female predominance (69.4%). Most patients (75.9%, *n* = 107) were employed. Median disease duration was 1.3 years (interquartile range [IQR] 0.8–2.1), and the median Expanded Disability Status score was 1.5 (IQR 0.0–2.0). Table 1 shows the main characteristics of the sample.

### 3.1. Awareness and Acceptance

Only 19.4% (*n* = 28) of patients had prior awareness of sNfL testing (Table 2). Following a standardized educational tutorial, acceptance rates were high, with 84.0% (*n* = 121) agreeing or strongly agreeing to use sNfL for disease monitoring.

Symptoms indicative of anticipatory anxiety prior to the appointment were reported by 30.3% (*n* = 44) of the cohort. Overall, 62.5% (*n* = 90) reported that they would feel more at ease while awaiting their follow-up visit if their sNfL levels were below the age-adjusted upper reference limit (Table 2).

### 3.2. Predictors of Acceptance

**Figure 1:**
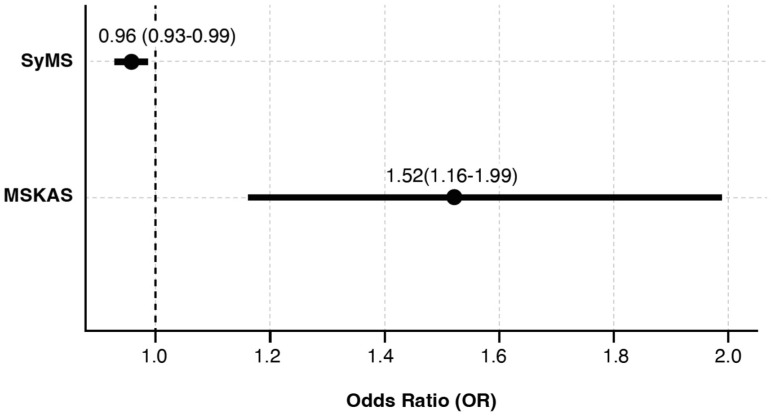
Predictors of acceptance. Note. Forest plot illustrating results from a multivariable logistic regression analysis (*n* = 144). The vertical line at an Odds Ratio (OR) = 1 denotes the null hypothesis (no association). MSKAS = Multiple Sclerosis Knowledge Assessment Scale; SyMS = SymptoMScreen questionnaire.

Patients willing to accept sNfL testing had higher knowledge of the disease, less treatment decision regret, better physical quality of life, and better SDMT performance in univariate comparisons (Table 1). Multivariable logistic regression analysis identified two independent predictors of greater acceptance: higher disease knowledge (OR = 1.52, 95%CI 1.16–1.99; *p* = 0.002) and a less severe symptom endorsement (OR = 0.96, 95%CI 0.93–0.99; *p* = 0.038) (Figure 1).

## 4. Discussion

The field of demyelinating diseases is undergoing a substantial transformation driven by the availability of high-efficacy disease-modifying therapies and the rapid advancement of fluid biomarkers [2,6,8,30]. Within the context of precision medicine, sNfL is recognized as a clinically relevant marker of neuroaxonal damage, providing objective, quantifiable data essential for personalized MS care [9,10,11,17]. Furthermore, shared decision-making has become a cornerstone of care, requiring the individualization of treatment based on clinical data, radiological indicators, and patient preferences [31,32,33].

Our study reports a high willingness (84.0% agreed or strongly agreed) to accept sNfL testing among patients with early RRMS and low physical disability after receiving a brief educational tutorial. This acceptance rate is consistent with the positive attitudes observed toward blood-based biomarkers for Alzheimer’s disease, reinforcing the general public’s receptiveness to objective, low-invasive neurodiagnostic tools [34,35].

The high acceptance was strongly linked to perceived psychological benefits: 62.5% of patients reported feeling significantly more at ease while awaiting routine follow-up if their sNfL levels were known to be within the normal limit. This finding is particularly salient given that anticipatory anxiety regarding the neurological appointment was reported by 30.3% of the cohort, and illness-related uncertainty is common in the MS population [1,3,4,36,37]. Uncertainty about prognosis, which only 41% of patients had discussed with their neurologist, is consistently associated with psychological distress [36]. Therefore, the high acceptance, driven by a desire to be “more at ease,” suggests sNfL is perceived as a valuable tool to objectively alleviate a patient’s uncertainty regarding the degree of disease control by providing complementary, quantifiable information on neuroaxonal damage between clinical visits.

In multivariable analysis, higher disease knowledge and lower symptom burden emerged as independent predictors of patient willingness to adopt sNfL testing. While baseline awareness of sNfL was low (19.4%), the rate of informed acceptance was high, catalyzed by a desire for objective data to mitigate the profound illness-related uncertainty inherent in MS. Patient engagement appears fundamentally linked to health literacy. Although MS populations often demonstrate suboptimal disease and risk knowledge, higher levels of such information are associated with improved medication adherence and a greater readiness to initiate therapy [38,39]. Factors such as advanced education, prior experience with disease-modifying therapies, and superior data interpretation skills correlate with higher risk knowledge, whereas a greater fear of disability (e.g., wheelchair dependency) is negatively associated [39]. Informed patients are better able to integrate complex medical evidence and embrace innovations that address the core problem of disease unpredictability. These findings underscore the critical role of targeted education in fostering patient acceptance of innovative monitoring tools. Interestingly, the association between lower symptom endorsement and greater acceptance contrasts with previous research suggesting that poor clinical status perception drives risk-seeking behavior toward high-efficacy treatments [40]. This discrepancy may indicate that patients with lower physical impact are more psychologically prepared to engage with proactive monitoring, perceiving a higher “capacity to respond” to results. Conversely, individuals experiencing severe symptomatic distress may prioritize immediate symptom management over long-term prognostic biomarkers [41].

Our study has several limitations. First, the cross-sectional design prevents us from establishing temporal relationships or causality between variables, such as whether high knowledge precedes or results from acceptance. Second, relying exclusively on self-reported measures introduces potential response bias regarding sensitive topics. Third, the cohort of patients with early-stage RRMS and low physical disability (median EDSS 1.5) limits the generalizability of these findings to the entire MS spectrum. Furthermore, we acknowledge a potential selection bias, as more motivated individuals may have enrolled. Beyond these study-specific factors, the clinical utility of sNfL is constrained by its lack of disease specificity, as it reflects general neuroaxonal injury and can be elevated in various other neurological conditions [8]. Its levels are also significantly influenced by physiological confounders such as age, Body Mass Index, and renal function [42,43]. Collectively, these hurdles, including age and comorbidity confounding, lesion-related variability, and the current lack of assay non-standardization, contribute to a delayed clinical integration of sNfL into the management of individual patients [43,44]. Consequently, while we found high willingness, the study did not assess the long-term sustainability of this acceptance or account for real-world barriers like healthcare system constraints, costs, or long-term adherence, including the unmeasured influence of the treating neurologist’s opinion [15,43,44].

## 5. Conclusions

The management of MS is rapidly evolving toward personalized therapeutic strategies that necessitate continuous, objective monitoring. Our findings provide essential patient-perspective information, demonstrating a robust acceptance rate for sNfL testing when supported by adequate educational context. This high level of receptivity emphasizes the necessity of anchoring precision medicine in the principles of shared decision-making. By incorporating patient preferences for objective surveillance and prioritizing targeted educational interventions to address existing knowledge deficits, multidisciplinary teams can improve overall health literacy and ensure that management is optimized according to each patient’s cognitive and psychological readiness. Ultimately, integrating sNfL into routine care facilitates a more proactive, patient-centered approach that aligns biological data with individual patient needs.

## Figures and Tables

**Table 1:** Main characteristics of the participants.

	Total *n* = 144	Willing to Accept sNfL Testing *n* = 121	Unwilling *n* = 23	*p*-Value
Age, years, mean (SD)	37.6 (10.3)	37.3 (10.3)	38.8 (10.6)	0.529
Sex, female, *n* (%)	100 (69.4)	82 (67.8)	18 (78.3)	0.317
Type of education, university, *n* (%)	63 (43.8)	55 (45.5)	8 (34.8)	0.453
Employed, *n* (%)	107 (75.9)	90 (75.6)	17 (77.3)	0.749
Disease duration, years, median (IQR)	1.3 (0.8–2.1)	1.3 (0.8–2.2)	1.3 (0.8–1.9)	0.717
Number of relapses, mean (SD)	1.5 (0.7)	1.4 (0.7)	1.7 (0.8)	0.131
EDSS score, median (IQR)	1.5 (0.0–2.0)	1.5 (0.0–2.0)	1.5 (0.0–2.0)	0.798
SyMS score, median (IQR)	10.0 (4.0–20.0)	9.0 (4.0–19.0)	12.0 (6.0–31.0)	0.092
HADS Anxiety score, mean (SD)	8.3 (5.3)	8.1 (5.3)	9.6 (5.7)	0.274
HADS Depression score, mean (SD)	4.5 (3.8)	4.3 (3.7)	5.9 (4.3)	0.090
BHS score, mean (SD)	4.1 (3.6)	3.8 (3.3)	5.5 (4.9)	0.232
MSIS-29, Physical score, median (IQR)	8.3 (1.7–30.0)	8.3 (0.8–27.5)	16.7 (7.5–45.0)	0.034
MSIS-29, Psychological score, median (IQR)	25.9 (11.1–48.1)	25.9 (11.1–48.1)	37.0 (14.8–75.9)	0.153
SDMT score, mean (SD)	49.9 (12.5)	50.8 (12.2)	44.9 (13.5)	0.045
MSKAS score, mean (SD)	18.4 (2.1)	18.7 (2.0)	16.8 (1.8)	<0.001
MUIS score, mean (SD)	28.3 (8.5)	27.8 (8.2)	31.3 (1.0)	0.109
LTP communication, yes, *n* (%)	59 (41.0)	52 (43.0)	7 (30.4)	0.262
DRS score, median (IQR)	10.0 (0.0–25.0)	5.0 (0.0–20.0)	25.0 (5.0–40.0)	<0.001
SURE < 4, *n* (%)	35 (26.5)	31 (28.2)	4 (18.2)	0.332

Note. BHS = Beck Hopelessness Scale; DRS = Decision Regret Scale; EDSS = Expanded Disability Status Scale; HADS = Hospital Anxiety and Depression Scale; IQR = Interquartile range; LTP = Long-term prognosis communication; MSIS-29 = Multiple Sclerosis Impact Scale; MSKAS = Multiple Sclerosis Knowledge Assessment Scale; MUIS = Mishel Uncertainty of Illness Scale; SD = Standard deviation; SDMT = Symbol Digital Modalities Test; SURE = 4-item Decisional Conflict Scale; SyMS = SymptoMScreen questionnaire.

**Table 2:** Knowledge and attitudes toward sNfL (*n* = 144).

(a) How much do you understand about how this biomarker based on serum NfL works? *n* (%)
1. I never heard about it: 87 (60.4)
2. I have heard about it, but I don’t understand it: 29 (20.1)
3. I have some understanding about it: 16 (11.1)
4. I understand quite well about it: 8 (5.5)
5. I understand it and I could explain it to others: 4 (2.8)
(b) After reading this information, would you accept the use of NfL in blood to monitor your disease activity? *n* (%)
1. Strongly agree: 88 (61.1), 2. Agree: 33 (22.9), 3. Neither agree nor disagree: 15 (10.4), 4. Disagree: 2 (1.4), 5. Strongly disagree: 6 (4.2)
(c) In the case of knowing the result of serum NfL < 10 pg/mL, would you feel more at ease while waiting for the next follow-up visit? *n* (%)
1. Yes: 90 (62.5), 2. No: 6 (4.2), 3. I don’t know: 48 (33.3)

Note. sNfL = Serum neurofilament light chain.

## Data Availability

The datasets generated during the analysis of the study are available from the corresponding author upon reasonable request.

## Appendix A

The following contextual information was provided before the initial assessment:

“Your condition is routinely monitored using magnetic resonance imaging (MRI) and follow-up testing (blood tests). Advances are currently being explored in new monitoring techniques, such as measuring neurofilament light chain (NfL) in the blood, a determination that could potentially provide more precise and less invasive information about MS status”.

(a) How much do you understand about how this biomarker based on serum NfL works? Please, select one option:
  (1) I never heard about it
  (2) I have heard about it, but I don’t understand it
  (3) I have some understanding about it
  (4) I understand quite well about it
  (5) I understand it and I could explain it to others

**Educational Tutorial Text**

“Neurofilaments (NfL) are proteins that are part of the cellular skeleton of neurons (the cytoskeleton). When there is cellular damage to these cells, NfL are released into the cerebrospinal fluid (CSF) and subsequently reach the blood. Their presence indicates ongoing axonal damage, suggesting significant deterioration of nerve cells and potentially more active disease. The generally accepted threshold for elevation is NfL > 10 pg/mL. Researchers are investigating this biomarker to provide clues about the long-term evolution of MS. It is already known to predict acute inflammatory phenomena, such as flares and short-term disability progression. The potential to predict the level of long-term disability is currently under investigation. The goal of measuring NfL in the blood is to enable the early detection of patients who may be at risk of MS worsening. This technique typically involves blood sampling every 3 to 6 months”.

**Post Tutorial Test**

(b) After reading this information, would you accept the use of NfL in blood to monitor your disease activity?
  (1) Strongly agree
  (2) Agree
  (3) Neither agree nor disagree
  (4) Disagree
  (5) Strongly disagree
(c) In the case of knowing the result of serum NfL <10 pg/mL, would you feel more at ease while waiting for the follow-up visit?
  (1) Yes
  (2) No
  (3) I don’t know
